# Usability of a Mobile App for Real-Time Assessment of Fatigue and Related Symptoms in Patients With Multiple Sclerosis: Observational Study

**DOI:** 10.2196/19564

**Published:** 2021-04-16

**Authors:** Miklos Palotai, Max Wallack, Gergo Kujbus, Adam Dalnoki, Charles Guttmann

**Affiliations:** 1 Center for Neurological Imaging, Department of Radiology Brigham and Women’s Hospital, Harvard Medical School Boston, MA United States; 2 Mobilengine Budapest Hungary

**Keywords:** multiple sclerosis, fatigue, depression, mobile application, mobile phone, real-time assessment

## Abstract

**Background:**

Although fatigue is one of the most debilitating symptoms in patients with multiple sclerosis (MS), its pathogenesis is not well understood. Neurogenic, inflammatory, endocrine, and metabolic mechanisms have been proposed. Taking into account the temporal dynamics and comorbid mood symptoms of fatigue may help differentiate fatigue phenotypes. These phenotypes may reflect different pathogeneses and may respond to different mechanism-specific treatments. Although several tools have been developed to assess various symptoms (including fatigue), monitor clinical status, or improve the perceived level of fatigue in patients with MS, options for a detailed, real-time assessment of MS-related fatigue and relevant comorbidities are still limited.

**Objective:**

This study aims to present a novel mobile app specifically designed to differentiate fatigue phenotypes using circadian symptom monitoring and state-of-the-art characterization of MS-related fatigue and its related symptoms. We also aim to report the first findings regarding patient compliance and the relationship between compliance and patient characteristics, including MS disease severity.

**Methods:**

After developing the app, we used it in a prospective study designed to investigate the brain magnetic resonance imaging correlates of MS-related fatigue. In total, 64 patients with MS were recruited into this study and asked to use the app over a 2-week period. The app features the following modules: Visual Analogue Scales (VASs) to assess circadian changes in fatigue, depression, anxiety, and pain; daily sleep diaries (SLDs) to assess sleep habits and quality; and 10 one-time questionnaires to assess fatigue, depression, anxiety, sleepiness, physical activity, and motivation, as well as several other one-time questionnaires that were created to assess those relevant aspects of fatigue that were not captured by existing fatigue questionnaires. The app prompts subjects to assess their symptoms multiple times a day and enables real-time symptom monitoring through a web-accessible portal.

**Results:**

Of 64 patients, 56 (88%) used the app, of which 51 (91%) completed all one-time questionnaires and 47 (84%) completed all one-time questionnaires, VASs, and SLDs. Patients reported no issues with the usage of the app, and there were no technical issues with our web-based data collection system. The relapsing-remitting MS to secondary-progressive MS ratio was significantly higher in patients who completed all one-time questionnaires, VASs, and SLDs than in those who completed all one-time questionnaires but not all VASs and SLDs (*P*=.01). No other significant differences in demographics, fatigue, or disease severity were observed between the degrees of compliance.

**Conclusions:**

The app can be used with reasonable compliance across patients with relapsing-remitting and secondary-progressive MS irrespective of demographics, fatigue, or disease severity.

## Introduction

### Background

Multiple sclerosis (MS) is an inflammatory, demyelinating disorder of the central nervous system that currently affects nearly 750,000 people in the United States [1]. Fatigue is one of the most disabling symptoms, affecting more than 65% of patients [2]. Fatigue has been associated with disease progression [3], and its pharmacological management remains challenging [4,5].

The pathogenesis of MS-related fatigue is not well understood. Neurogenic, inflammatory, endocrine, and metabolic mechanisms have been proposed [6]. In addition, several other factors, such as comorbid depression, anxiety, and sleep abnormalities, as well as physical activity and medications, may interfere with the perceived level of fatigue [2,6]. In fact, different fatigue phenotypes may exist in different patients. The ability to distinguish patient phenotypes might impact the understanding of underlying mechanisms and enable personalized management of this debilitating symptom. As an example of the need to capture the circadian nature of mood symptoms, including fatigue, it has been suggested that most people may experience lower energy levels in the evening than in the morning [7], whereas in a subset of patients affected by major depressive disorder, mood symptoms might be worse in the morning than in the evening [8]. In addition, in a recent study, we demonstrated the advantages of classifying patients according to temporal patterns of fatigue when associating damage to select brain circuitries with fatigue in patients with MS [9-14]. Although these studies highlighted the relevance of assessing temporal patterns of fatigue, retrospective data only included long-interval (every 1-2 years), repeated measures of fatigue, which might not reflect pathophysiologically relevant patterns.

### Objectives

We developed a mobile app to enable circadian assessment of fatigue and other mood symptoms, with the longer-term goal of identifying clinically and pathophysiologically relevant phenotypes of fatigue. As an example of its potential significance, this mobile app will enable us to test the hypothesis that diverse fatigue phenotypes may respond to different mechanism-specific treatments. Although several drugs have demonstrated efficacy in improving wakefulness in other conditions (such as narcolepsy [15]), none have been proven effective in treating fatigue in MS [4,5].

Several tools have been developed to assess various symptoms (including fatigue), monitor clinical status [14,16-20], or improve the perceived level of fatigue in patients with MS [16,19,21-23]. We believe that the presented mobile app meets a specific need for a tool to characterize fatigue phenotypes in MS by assessing the temporal patterns of fatigue and its comorbid mood symptoms.

In this study, we aim to test and use this mobile app in the framework of a prospective study that was designed to investigate the association between MS-related fatigue and structural brain damage. The overarching goal of this study is to identify brain magnetic resonance imaging (MRI) predictors of persistent and treatment-resistant fatigue in MS. The aim of this study is to describe the design of the mobile app and to report the first findings regarding patient compliance and the relationship between compliance and patient characteristics, including MS disease severity.

## Methods

### Study Population and Study Design

Patients with MS were recruited from the Comprehensive Longitudinal Investigation of Multiple Sclerosis at Brigham and Women’s Hospital (CLIMB) study [24] (n>2400), a large-scale, long-term study of patients with MS, using the following selection criteria: (1) availability of at least one previously recorded Modified Fatigue Impact Scale (MFIS) [25].

r Quality of Life in Neurological Disorders (Neuro-QoL) [26] score, (2) brain MRI scan acquired within 1 month of recruitment into the study, and (3) absence of clinical exacerbation within 3 months before their MRI. Selected patients were mailed a recruitment letter at least 2 weeks before their scheduled clinical visit at Brigham and Women’s Hospital (BWH). The letter contained a brief description of our study procedures and invited patients who were interested in participating in our research study for an in-person study initiation session (SIS) following their scheduled clinical appointment or at another scheduled time at BWH. The aim of the SIS was to (1) provide patients with a detailed description of our study; (2) obtain a written informed consent form; and (3) teach patients how to use the following 3 study devices: the abovementioned mobile app installed on a mobile device, a wrist-worn actigraphic MotionLogger watch [27], and a Nox T3 home sleep test (HST) device [28]. The actigraphic watch assessed physical activity during the daytime and sleep quality at night throughout the entire study (ie, for 2 weeks), whereas the HST device was used to assess sleep apnea and periodic limb movements at one night in the patient’s home. Data collected using the actigraphic watch and the HST device are not presented in this paper. Between May 2018 and September 2019, 64 patients with MS were recruited into the study and provided written informed consent in accordance with our study protocol approved by the institutional review board of BWH. Demographic data of these patients are presented in Table 1. Participants who answered all questions and returned all study devices to our laboratory received a remuneration of US $100.

**Table 1:** Characteristics and compliance of the study participants.

Variable			All patients		Group 1: completed all one-time questionnaires, VASs^a^, and SLDs^b^		Group 2: completed all one-time questionnaires but not all VASs and SLDs		Group 3: answered some one-time questionnaires but no VAS or SLD		Group 4: did not answer any one-time questionnaire, VAS, or SLD
Subjects, n (%)			64 (100)		47 (73)		4 (6)		5 (8)		8 (13)
Age (years), mean (SD)			52 (9)		52 (8)		54.4 (10)		44.6 (10)		55.1 (8)
Sex (female), n (%)			54 (84)		40 (85)		4 (100)		5 (100)		4 (50)
Non-White or Hispanic, n (%)			3 (5)		2 (4)		0 (0)		1 (20)		0 (0)
**Disease category, n (%)**											
	Relapsing-remitting multiple sclerosis	58 (100)		46 (79)		2 (3)		3 (5)		7 (12)	
	Secondary-progressive multiple sclerosis	5 (100)		1 (20)		2 (40)		1 (20)		1 (20)	
	Clinically isolated syndrome	1 (100)		0 (0)		0 (0)		1 (100)		0 (0)	
Disease duration (years), mean (SD)			20 (8)		21 (8)		28 (10)		14 (6)		19 (7)
Expanded Disability Status Scale (years), mean (SD)			2.1 (1.5)		2.0 (1.1)		3.0 (2.4)		2.2 (2.5)		2.7 (2.3)
Fatigue Severity Scale score, mean (SD)			42 (13)^c^		41 (13)		47 (12)		43 (15)^d^		N/A^e^

^a^VASs: Visual Analogue Scales.

^b^SLDs: sleep diaries.

^c^In total, 55 patients completed the Fatigue Severity Scale. Therefore, the mean (SD) score of the Fatigue Severity Scale was calculated using 55 patients’ data.

^d^One patient did not complete the Fatigue Severity Scale in this group. Therefore, the mean (SD) score of the Fatigue Severity Scale in this group was calculated using 4 patients’ data.

^e^N/A: not applicable.

### Design of the Mobile App

Our research team, comprising physician scientists, designed and developed a mobile app in collaboration with the software developer team of *Mobilengine* [29]. Our teams communicated through virtual workshops. The app was installed on an Android smartphone with a 5-inch high-definition display that was provided to the participants. The participants used the smartphone for 2 weeks, during which they followed their normal daily routine. They then returned their smartphones to our research laboratory. The mobile app features 3 modules, which we describe in detail below, and in Multimedia Appendix 1: (1) a series of one-time questionnaires, to be answered within 3 days of enrollment (Table 2); (2) Visual Analogue Scales (VASs) for self-reporting of fatigue, depression, anxiety, and pain levels, every 4 hours while awake; (3) a sleep diary (SLD) with separate items to be completed before and after each sleep or nap, containing a series of questions regarding perceived duration and quality of sleep, as well as the same VAS described earlier. VAS and SLD data were consistently collected for 14 days, starting after all one-time questionnaires were answered. Therefore, the duration of the data collection varied between 14 and 17 days depending on how much time it took for the participant to answer all one-time questionnaires. Of note, subjects were allowed to pause their entries and return to complete the remainder of the questions at a later time.

**Table 2:** Summary of existing questionnaires incorporated into our one-time questionnaire module.

Questionnaire	Domain assessed	Study
The Fatigue Severity Scale	Fatigue	Krupp et al (1989) [30]
The Neuro-QoL^a^ fatigue questionnaire	Fatigue	Cella et al (2012) [26]
The Neuro-QoL depression questionnaire	Depression	Cella et al (2012) [26]
The Neuro-QoL anxiety questionnaire	Anxiety	Cella et al (2012) [26]
The Neuro-QoL sleep questionnaire	Sleep quality	Cella et al (2012) [26]
The Modified Fatigue Impact Scale	Fatigue	Amtmann et al (2012) [25]
Symptoms of depression questionnaire (7 questions only)	Vegetative symptoms of depression	Pedrelli et al (2014) [31]
The Epworth Sleepiness Scale	Sleepiness	Johns (1991) [32]
The Godin Leisure-Time Exercise questionnaire	Physical activity	Godin (1985) [33]
The Behavioral Approach System and Behavioral Avoidance System scale	Drive; fun seeking; reward responsiveness	Carver and White (1994) [34]

^a^Neuro-QoL: Quality of Life in Neurological Disorders.

The following measures were taken to prevent slip errors: (1) user control and freedom: the selected (or entered) answer was submitted only when the user tapped the forward (or backward) arrow on the screen; (2) flexibility: users can freely navigate forward and backward between questions within the same block (ie, the same questionnaire) and make corrections as needed; (3) consistent and standard user interface: every scale had the same size and all questions, answer options, and forward and backward arrows were located on the same part of the screen. In addition, the app also has a landscape mode, in which the distance between the points of the VAS is larger; and (4) visibility and system status: the number of completed questions out of the total number of questions was shown at the bottom of the screen.

#### One-Time Questionnaire Module

**Figure 1:**
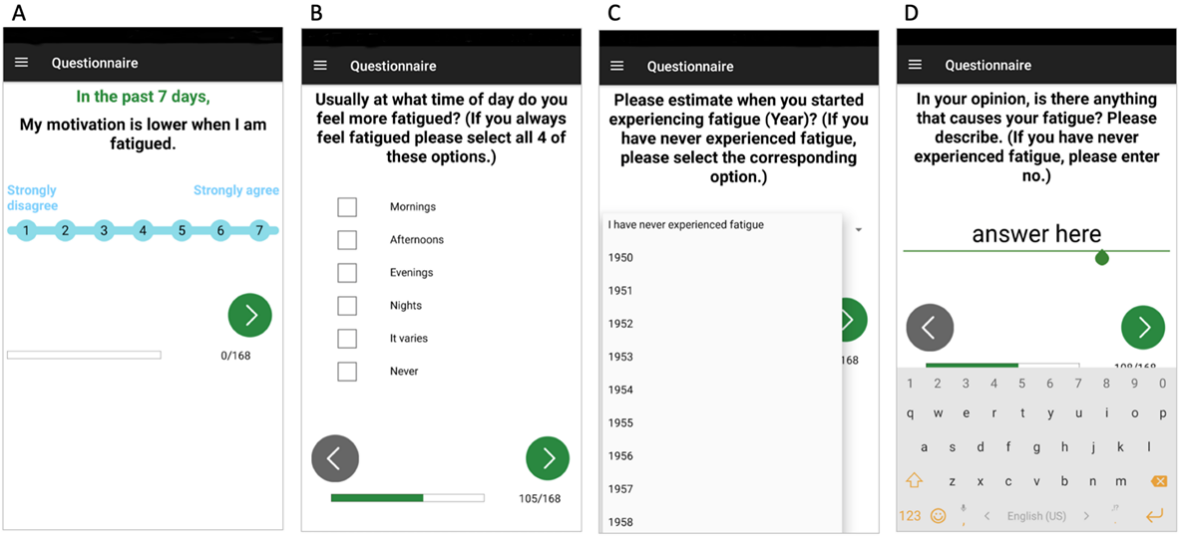
Sample screens for simple choice (A), multiple choice (B), number box (C), and text box (D) questions used in the one-time questionnaire module. Users can freely navigate forward and backward between questions within the same block using the forward (green) and backward (gray) arrows at the bottom of the screen. Under the arrows, a status bar indicates how far through the one-time questions a user is.

We included 10 questionnaires (Table 2) that were previously validated. In addition, we created 22 new questions to assess the relevant aspects of fatigue that were not captured by existing fatigue questionnaires, such as (1) circadian differences in fatigue severity and (2) effects of caffeine and nicotine use on the perceived level of fatigue, and (3) we incorporated open-ended questions aimed at exploring other potential aggravators and alleviators of fatigue (questions are presented on pages 16-20 of Multimedia Appendix 1). The one-time questionnaire module consisted of 168 questions. We preserved wording and the type of response (single choice, multiple choice, free text, analog scale, and selection of date) applied in the original questionnaires for each question included in our app (Figure 1). Questions were grouped into separate *blocks*, reflecting the original questionnaires. Patients were presented 1 question at a time but were able to freely navigate forward and backward between questions within the same block. Participants were notified at the end of each block and cautioned to be confident with their previous responses before final submission because they would no longer be able to return to a previous block of questions.

#### VAS Module

**Figure 2:**
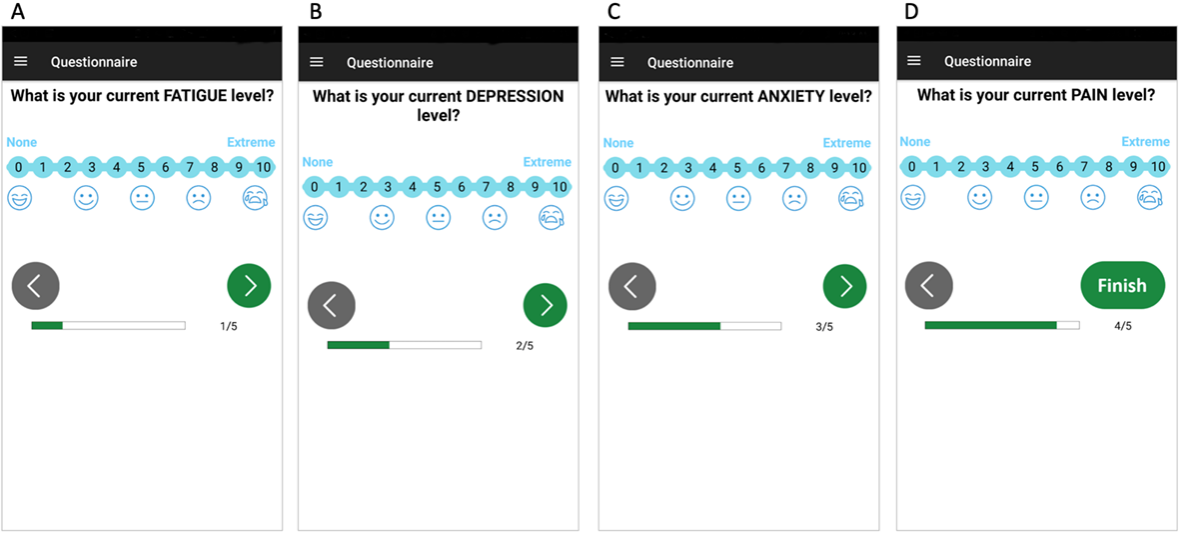
Sample screens for Visual Analogue Scales for fatigue (A), depression (B), anxiety (C), and pain (D). Users can freely navigate forward and backward between questions using the forward (green) and backward (gray) arrows at the bottom of the screen. Under the arrows, a status bar indicates the progress of the user in the Visual Analogue Scale.

The VAS [35-39] module was used to assess a subject’s current level of fatigue, anxiety, depression, and pain. Each of these 4 VASs is scored between 0 and 10, with 0 representing absence (*none*) of the symptom (eg, *no fatigue*) and 10 representing *extreme* presence of the symptom (Figure 2). Previous studies have shown a very high correlation between VAS and a series of line drawings of faces with expressions of increasing distress [40,41]. We added a *faces* scale under each VAS under the assumption that the combination of the 2 scales may yield more robust results. Reminder functions were implemented in the mobile app based on the results of previous studies that associated reminders with positive effects on engagement with digital behavior change interventions [42]. One of our main aims was to assess circadian changes in fatigue, anxiety, depression, and pain by measuring the level of these symptoms at least once in the morning, once in the afternoon, and once in the evening or night. Therefore, while awake, patients were prompted every 4 hours to complete the 4 VASs. Prompting was achieved through an acoustic and haptic (vibratory) alarm as well as a visual notification. In the absence of a response, the alarm and notification would be presented again after 30, 60, 90, and 120 minutes. If a subject did not answer a VAS within the allotted 2-hour period (between 4 and 6 hours after the previous VAS was completed), the subject would not be able to complete the VAS since beyond the allotted 2-hour period, the pending measurement would be closer in time to the next scheduled VAS assessment. Rather, they would be presented with notifications for a new VAS during an additional 2 hours (8 hours after the previous VAS had been completed).

#### SLD Module

**Figure 3:**
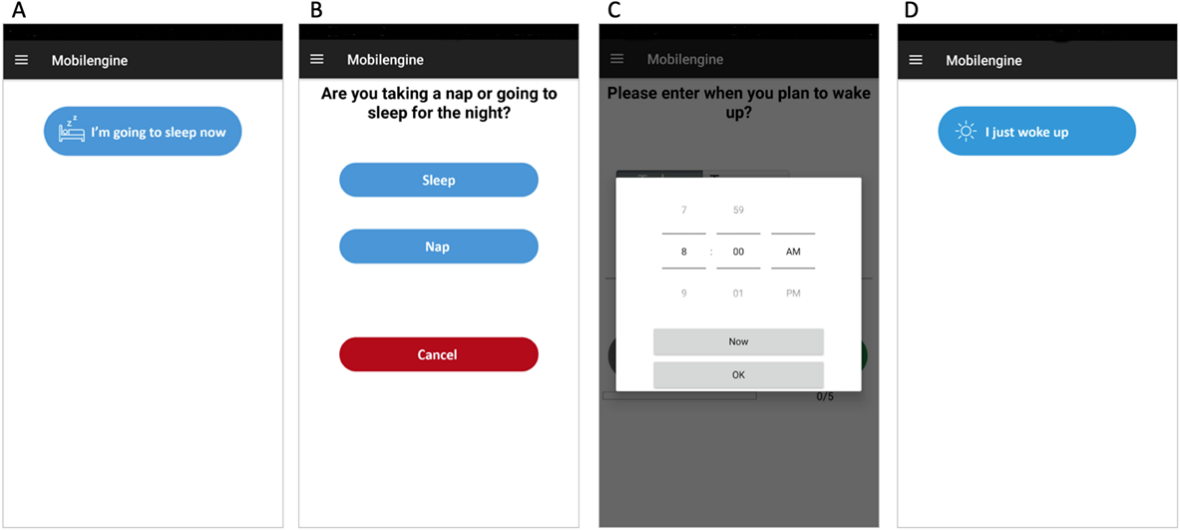
Sample screens for the sleep diary module. Sleep diary is activated by tapping on the “I’m going to sleep now” icon (A). Then, the user is prompted to indicate whether they were taking a nap or going to sleep for the night (B), as well as their intended wake-up time (C). Upon awakening, the user selects the “I just woke up” icon (D).

**Figure 4:**
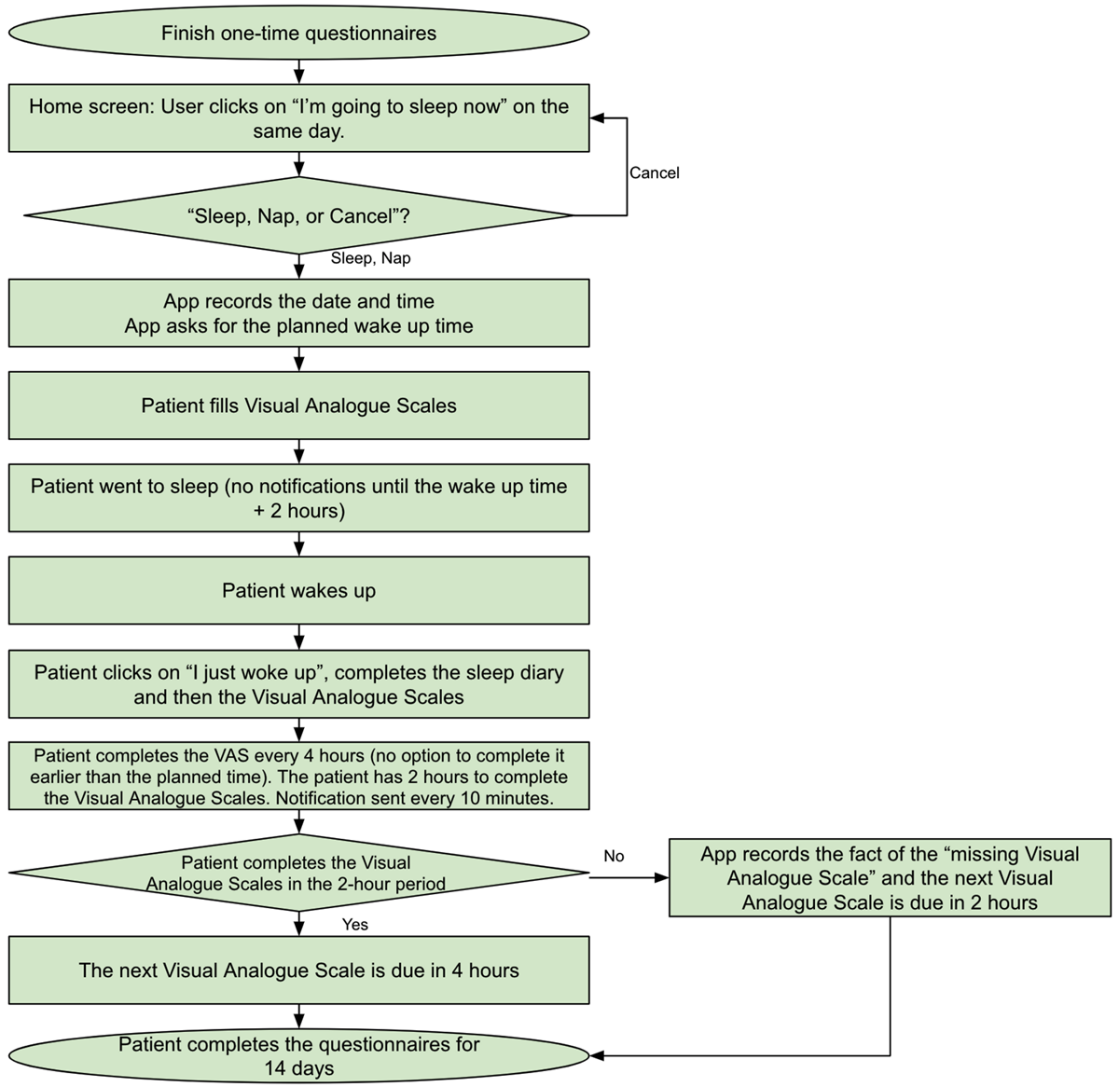
Algorithm of Visual Analogue Scale and sleep diary assessments. VAS: Visual Analogue Scale.

The SLD contains items to be completed before sleep and items required after awakening from night sleep or daytime naps. At the time of recruitment, patients were instructed to activate the SLD immediately before any planned sleep period. Before sleep, the patient was prompted to indicate whether they were taking a nap or going to sleep for the night and their intended wake-up time (Figure 3). Then, the 4 VASs were presented to the patient for completion before the sleep episode. Sound alarms and notifications were deactivated in the mobile app until 2 hours following the intended wake-up time provided by the patient. Patients were at liberty to wake up spontaneously or prompted by a preprogrammed device, such as an alarm clock. On awakening, the patient was required to select the “I just woke up” icon on their phone (Figure 3). If this option was not activated 2 hours after the intended wake-up time, sound, haptic, and visual alerts were activated. The “I just woke up” icon gives access to the wake items of the SLD that were developed by the Division of Sleep Medicine at BWH [43] and assesses final wake time, time awake before scheduled rising time, sleep latency, number of awakenings, sleep duration, subjective sleep quality, as well as current feeling of refreshed, sleepiness, and tenseness (wake items of the SLD are presented on page 21 of Multimedia Appendix 1). These items were followed by the day’s first VAS. Figure 4 shows the algorithm for the SLD and VAS assessments. If no sleep or nap option was selected on a given day, subjects were prompted by the app at 4 AM of the following calendar day, “We noticed that you did not indicate when you went to sleep last night. Please select the appropriate answer below:” with options for “I forgot to indicate when I went to sleep” and “I did not go to sleep.” If “I forgot to indicate when I went to sleep” was selected, the participant was prompted to enter an answer for “Time into bed” and to fill out an SLD.

The sequence of the questionnaires and questions was the same every time. All questions had to be answered before the questionnaire was submitted. At the bottom of the screen, a status bar indicated progress of the participant in the one-time questionnaires or VASs (Figures 1 and 2). The opening and submission dates and times were recorded for each question. All participants received an anonymized unique subject ID. No other identifying information was entered into the app.

#### Monitoring the Database

All functions of the app were available offline (ie, with no internet connection) without limitations. The app saved and stored all subject responses locally in the smartphone’s internal storage space. Each smartphone was provided with Wi-Fi and 3G (Third Generation) internet access to upload the collected data to Mobilengine’s designated server as soon as each item was completed. If the network connection was inconsistent, the data were transferred as soon as the connection was adequate. This system allowed us to monitor data collection in real time on demand. In addition, an email was sent daily at 4 PM Eastern time to designated investigators, listing the currently active devices with their IDs, as well as either (1) the progress of the subject in the one-time questionnaires or (2) whether the subject had filled out at least one VAS or SLD during the previous 24 hours. Subjects who did not complete their daily tasks are highlighted in red.

Following the completion of 14 days of VASs and SLDs, the subject’s monitoring period was concluded, and they no longer had the option to answer further questions. The subject code was removed from daily emails. The ability to monitor our subjects’ responses in this way allowed the investigators to promptly notify subjects who were not completing the study in the recommended time frame rather than waiting for the mobile device to be returned at the end of a 14- to 17-day period. We consider this monitoring feature to be an important part of mobile apps. All results in the database were easily exportable as a table with rows containing subject ID, individual question ID, individual question response, and time of response. Every answer or item created a new row in the table (ie, a long data format).

### Assessment of Patient Compliance

Patient compliance was defined as adherence to the study protocol and was measured by the number of completed questions. We aimed to assess the association between the number of completed questions and demographic and clinical data. To this end, we grouped the recruited patients into the following 4 *compliance* groups: group 1: completed all one-time questionnaires, VASs, and SLDs; group 2: completed all one-time questionnaires but did not answer all VASs and SLDs; group 3: answered some, but not all one-time questionnaires and did not answer any VAS or SLD; group 4: did not answer any one-time questionnaire, VAS, or SLD. These groups were defined after the completion of patient recruitment. The following variables were compared between the 4 groups: age, sex, ethnicity, disease category (ie, relapsing-remitting multiple sclerosis [RRMS] and secondary-progressive multiple sclerosis [SPMS] ratio), disease duration, and physical disability (assessed using the Expanded Disability Status Scale [44]). These variables were assessed as part of the patients’ routine clinical visits. We also compared the Fatigue Severity Scale (FSS) [30] score between the abovementioned groups 1 to 3 (group 4 was not included in this analysis because patients in group 4 did not complete any questions) to assess whether baseline fatigue level was associated with patient compliance.

### Statistical Analysis

Differences in demographic and clinical variables between the patient compliance groups were assessed using analysis of variance or Kruskal-Wallis tests for continuous variables (depending on the distribution of the data) and the chi-square test or Fisher exact test (when n<5) for categorical variables. The threshold for statistical significance was set at *P*<.05. All statistical analyses were performed using Stata Statistical Software: Release 13 (StataCorp).

## Results

### Patient Compliance

**Figure 5:**
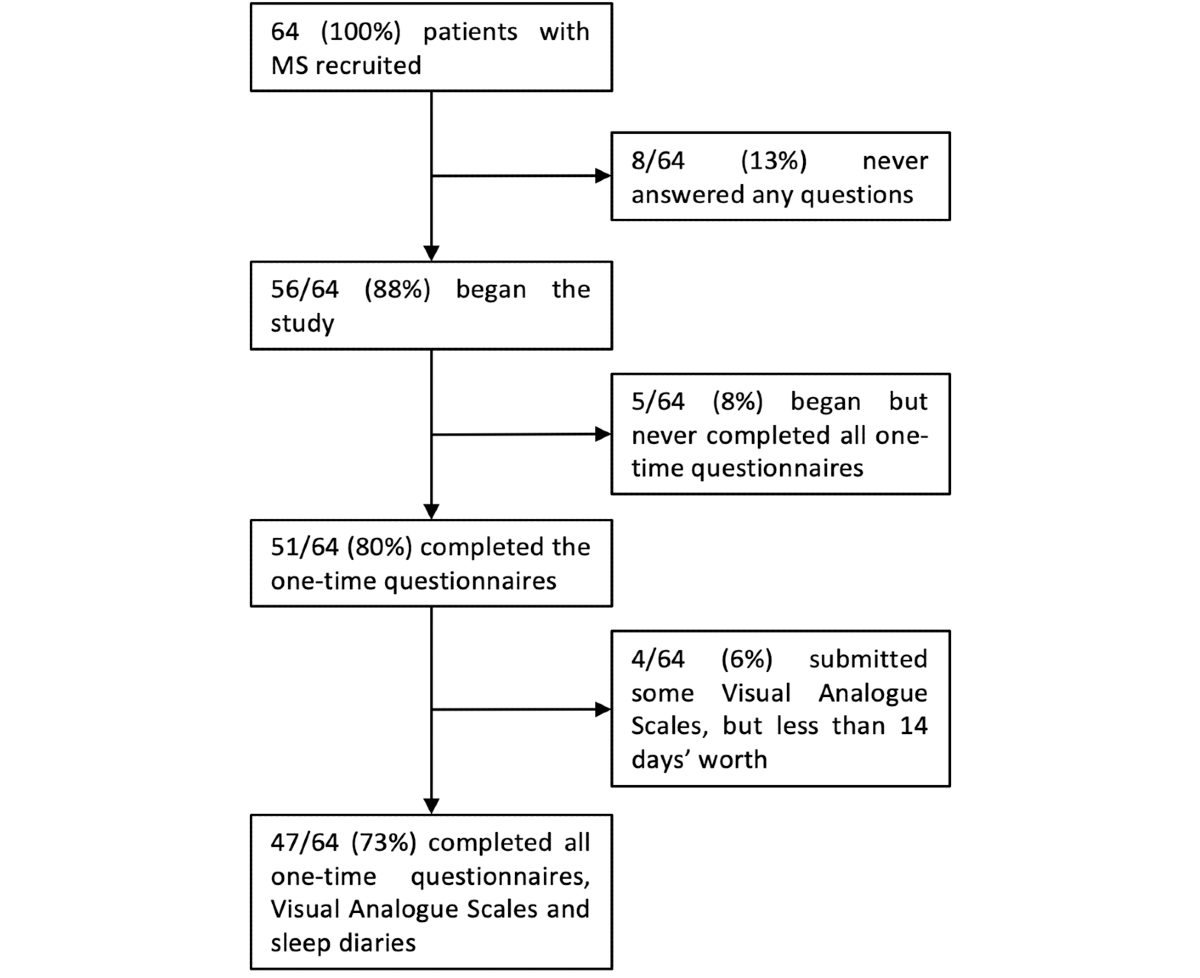
Patient compliance in our study cohort. MS: multiple sclerosis.

Of the 64 patients, 56 (88%) began the study (ie, answered at least one question), whereas 8 (13%) patients did not complete any questions (Figure 5). Of the 56 patients who began the study, 51 (91%) completed the one-time questionnaires and 47 (84%) completed both the one-time questionnaire as well as the 14-day VAS and SLD modules (Figure 5).

**Figure 6:**
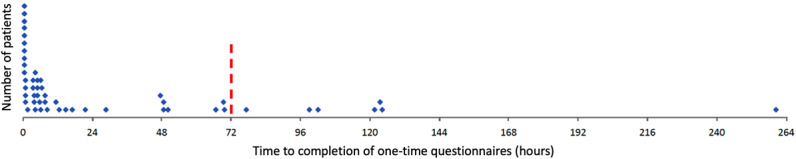
Time to completion of one-time questionnaires (expressed in hours). Patients (indicated by blue rhombuses) were asked to answer all one-time questions within 3 days of enrollment (indicated by the dashed vertical red line).

Among the 51 out of 64 patients who completed the one-time questionnaires, 44 (86%) met the target of 72 hours, and the median time to completion was 6.19 hours, with a range from 21 minutes to 10.9 days (Figure 3). Of the 51 patients, 14 (28%) completed the one-time questionnaires within an hour, 20 additional patients (39%) completed it on the same day that they started it, and 5 additional patients (10%) completed the questionnaire within a 48-hour period (Figure 6).

All subjects who completed the one-time questionnaire module also completed at least 2 days of VAS modules. Of the 51 subjects, 3 (6%) stopped after 8 days or less.

Study subjects submitted close to 4 VASs per day with a mean of 3.9 (SD 1.3).

### Demographic and Clinical Comparison of Compliance Groups

There were no significant differences in age, sex, ethnicity, disease duration, and Expanded Disability Status Scale scores between the 4 patient compliance groups (Table 1). The RRMS/SPMS ratio was significantly higher in group 1 than in group 2 (*P*=.01; Table 1). There was no significant difference in the RRMS/SPMS ratios between the other groups. There was no significant difference in FSS scores between groups 1 to 3.

## Discussion

### Principal Findings

We developed and used a mobile app that is innovative because of the following aspects: (1) assessment of fatigue and its associated mood symptoms (ie, depression and anxiety) and pain using the VAS every 4 hours while patients are awake for 2 weeks; (2) patient prompting to assess their symptoms multiple times a day; (3) real-time symptom monitoring of patients with MS by researchers or treating physicians through a web-accessible portal; and (4) single time point assessments of fatigue and its significant confounders, such as depression, anxiety, physical activity, sleep problems, and motivation level by commonly used questionnaires along with several other questions that we created to assess the relevant aspects of fatigue that were not captured by existing fatigue questionnaires. Here, we report good patient compliance with our mobile app–based assessments, that is, 91% (51/56) completed all one-time questionnaires and 84% (47/56) completed all one-time questionnaires, VASs, and SLDs out of those patients who started using the app (n=56). Patients reported no issues with the usage of the app, and there were no technical issues with our web-based data collection system.

### Limitations

The limitations of the study are as follows: (1) we did not systematically collect feedback about the app itself, its ease of use, or any patient suggestions about its features. However, our coinvestigators asked patients regarding their user experience and satisfaction when they contacted us (eg, because of the questions on the study protocol or the use of the study devices) or when our coinvestigators contacted the patients (eg, when the daily, system-generated email showed that a patient did not complete all questions on that day). Our high completion rate (47/56, 84% of those who began the study completed it) shows that many found it navigable and usable for the 2-week monitoring period. (2) The data collection and monitoring period was only 2 weeks. The observed patient compliance may change if the mobile app is used for a longer period (ie, beyond the novelty period) [45]. (3) The mobile app was installed on a smartphone that was provided to each participant. The use of a new device might have influenced the participants’ behavior. (4) The current version of our app was not gamified. We may consider using gamification strategies when developing the next version of the app to further increase patient compliance and improve user experience.

### Comparison With Previous Work

Recently published studies underlined the relevance of delivery of health care through mobile devices (ie, mobile health) in MS [46,47]. Although several tools have been developed to assess various symptoms (including fatigue) and monitor clinical status [14,16-20,48] or to improve the perceived level of fatigue in MS [16,19,21-23], our app is the first that was specifically designed to conduct a state-of-the-art characterization of MS-related fatigue, including high-frequency fatigue assessments and real-time symptom monitoring.

To assess fatigue, 3 different self-assessment questionnaires (ie, MFIS [25], FSS [30], and Neuro-QoL fatigue [26]) and repeated daily VAS were implemented in our app. The MFIS has cognitive, physical, and psychosocial domains and addresses the effect of fatigue on daily activities [49]. The FSS addresses mainly physical fatigue with only one question related to cognitive fatigue [30]. The add-on value of the Neuro-QoL fatigue questionnaire is that it uses semantics to better characterize fatigue (eg, “felt exhausted,” “had no energy,” “felt fatigued,” or “felt tired”). Both FSS and Neuro-QoL assess fatigue in the past week, whereas MFIS refers to fatigue in the past 4 weeks. In addition, we formulated several new questions to assess the relevant aspects of fatigue that were not captured by existing fatigue questionnaires (eg, circadian differences in fatigue severity as well as potential aggravators and alleviators of fatigue).

Mood symptoms (ie, depression and anxiety) are highly intercorrelated with fatigue in MS [50,51], and their prevalence is over 20% in MS [52]. However, the relationship between MS-related fatigue and depression is not well understood [6,53]. Clinical symptomatology and, accordingly, clinical assessments or diagnosis of fatigue and depression show considerable overlap, as exemplified by (1) *fatigue or loss of interest* and *diminished ability to think or concentrate, or indecisiveness* are part of the Diagnostic and Statistical Manual of Mental Disorders, Fifth Edition, diagnostic criteria of major depression; (2) *tiredness or fatigue*, *loss of energy,* and *concentration difficulty* are items on the Beck Depression Inventory [54]; and (3) impairment in concentration, thinking, and decision making are items in the MFIS [25]. Overlap in the clinical symptomatology of fatigue and depression may raise the question of whether these symptoms reflect separate entities with different etiologies and pathophysiologies or share a similar pathogenesis. To investigate the association between fatigue and depression and anxiety, we included two one-time questionnaires (ie, Neuro-QoL depression and anxiety batteries [26]) in our app along with daily VAS for depression and anxiety. In addition, we included those questions from the Symptoms of Depression questionnaire [31], which specifically assesses vegetative symptoms of depression under the hypothesis that the presence of vegetative symptoms distinguishes clinically significant depression from phenotypes of fatigue that are not associated with depression.

Other relevant confounders of fatigue include sleep abnormalities [6], physical activity [55], and motivation [56]. Patients with MS have significantly more sleep disturbances than the general population [6]. The prevalence of sleep abnormalities is approximately 50% in patients with MS [6]. To assess sleepiness and alterations in sleep quality, a one-time questionnaire (ie, Epworth sleepiness scale [32]) and daily SLD were incorporated into our mobile app.

Patients with MS may have a sedentary lifestyle because of associated disabilities (eg, muscle deconditioning or pain), diminished physical endurance, and deterioration of symptoms that could follow physical exertion [55]. However, a growing body of evidence suggests that nonpharmacological approaches (eg, exercise or psychobehavioral interventions) might be more effective in reducing MS-related fatigue than medications [57]. We included a one-time questionnaire (ie, Godin Leisure-Time Exercise Questionnaire [33]) to assess physical activity and created targeted questions to assess the effect of physical activity, occupational, and psychological therapies on the patients’ perceived level of fatigue.

The central fatigue model of Chaudhuri and Behan [58] hypothesized that central fatigue is caused by damage to the nonmotor components of the cortico-striato-thalamic system. Indeed, a more recent study associated MS-related fatigue with alterations in reward responsiveness [59]. Furthermore, several neuroimaging studies have found damage to reward-related brain regions in fatigued patients with MS [2,56]. Therefore, our app includes a self-assessment questionnaire (ie, behavioral approach system or behavioral avoidance system scale [34]) to measure reward responsiveness, drive, and fun seeking.

Previous studies have shown that fatigue fluctuates over time, even throughout the day [13,14]. Temporal fluctuations of fatigue are understudied in MS. Our recently published studies showed that taking into account temporal fatigue dynamics may improve the characterization of brain pathological correlates of MS-related fatigue in MS [9-11,60]. We included 4 VASs in our mobile app to assess circadian changes in fatigue, depression, anxiety, and pain levels under the hypothesis that the circadian evolution of these symptoms may show different patterns, potentially representing different fatigue phenotypes with different etiologies and pathogeneses.

To increase patient compliance, most of the abovementioned apps have built-in reminder functions, similar to our mobile app. In addition, our system provides real-time monitoring of data collection for researchers and patient-treating physicians. None of the abovementioned apps or platforms provided real-time monitoring of data collection.

### Conclusions

We developed a mobile app with reasonable patient compliance to assess fatigue and its confounders in patients with MS. This tool contains a battery of commonly used questionnaires and has the capability to monitor the level of subjective symptoms, such as fatigue, anxiety, depression, and pain, through VAS. We hope that it will facilitate remote monitoring of symptoms and adverse events and may allow more timely intervention than is possible with scheduled face-to-face visits.

## Appendix

Multimedia Appendix 1 One-time questionnaire module: 11 previously validated questionnaires were implemented in the mobile app along with several additional questions that we formulated to assess relevant aspects of fatigue that were not captured by the existing questionnaires (eg, diurnal differences in fatigue severity, as well as potential aggravators and alleviators of fatigue).

## Abbreviations

BWH: Brigham and Women’s Hospital
CLIMB: Comprehensive Longitudinal Investigation of Multiple Sclerosis at Brigham and Women’s Hospital
FSS: Fatigue Severity Scale
HST: home sleep test
MFIS: Modified Fatigue Impact Scale
MRI: magnetic resonance imaging
MS: multiple sclerosis
Neuro-QoL: Quality of Life in Neurological Disorders
RRMS: relapsing-remitting multiple sclerosis
SIS: study initiation session
SLD: sleep diary
SPMS: secondary-progressive multiple sclerosis
VAS: Visual Analogue Scale

## References

[1] Wallin MT, Culpepper WJ, Campbell JD, Nelson LM, Langer-Gould A, Marrie RA, Cutter GR, Kaye WE, Wagner L, Tremlett H, Buka SL, Dilokthornsakul P, Topol B, Chen LH, LaRocca NG (2019). The prevalence of MS in the United States. Neurology.

[2] Bakshi Rohit (2003). Fatigue associated with multiple sclerosis: diagnosis, impact and management. Mult Scler.

[3] Cavallari M, Palotai M, Glanz BI, Egorova S, Prieto JC, Healy BC, Chitnis T, Guttmann CR (2016). Fatigue predicts disease worsening in relapsing-remitting multiple sclerosis patients. Mult Scler.

[4] Miller P, Soundy A (2017). The pharmacological and non-pharmacological interventions for the management of fatigue related multiple sclerosis. J Neurol Sci.

[5] Yang TT, Wang L, Deng XX, Yu G (2017). Pharmacological treatments for fatigue in patients with multiple sclerosis: a systematic review and meta-analysis. J Neurol Sci.

[6] Induruwa I, Constantinescu CS, Gran B (2012). Fatigue in multiple sclerosis - a brief review. J Neurol Sci.

[7] Wood C, Magnello ME (1992). Diurnal changes in perceptions of energy and mood. J R Soc Med.

[8] Courtet P, Olié E (2012). Circadian dimension and severity of depression. Eur Neuropsychopharmacol.

[9] Palotai M, Cavallari M, Healy BC, Guttmann CR (2020). A novel classification of fatigue in multiple sclerosis based on longitudinal assessments. Mult Scler.

[10] Palotai M, Cavallari M, Koubiyr I, Pinzon AM, Nazeri A, Healy BC, Glanz B, Weiner HL, Chitnis T, Guttmann CR (2020). Microstructural fronto-striatal and temporo-insular alterations are associated with fatigue in patients with multiple sclerosis independent of white matter lesion load and depression. Mult Scler.

[11] Palotai M, Nazeri A, Cavallari M, Healy BC, Glanz B, Gold SM, Weiner HL, Chitnis T, Guttmann CRG (2019). History of fatigue in multiple sclerosis is associated with grey matter atrophy. Sci Rep.

[12] Palotai M, Guttmann CR (2020). Brain anatomical correlates of fatigue in multiple sclerosis. Mult Scler.

[13] Johansson S, Ytterberg C, Hillert J, Widén Holmqvist L, von Koch L (2008). A longitudinal study of variations in and predictors of fatigue in multiple sclerosis. J Neurol Neurosurg Psychiatry.

[14] Powell DJH, Liossi C, Schlotz W, Moss-Morris R (2017). Tracking daily fatigue fluctuations in multiple sclerosis: ecological momentary assessment provides unique insights. J Behav Med.

[15] Billiard M, Broughton R (2018). Modafinil: its discovery, the early European and North American experience in the treatment of narcolepsy and idiopathic hypersomnia, and its subsequent use in other medical conditions. Sleep Med.

[16] Alexander S, Peryer G, Gray E, Barkhof F, Chataway J (2020). Wearable technologies to measure clinical outcomes in multiple sclerosis: a scoping review. Mult Scler.

[17] Bove R, White CC, Giovannoni G, Glanz B, Golubchikov V, Hujol J, Jennings C, Langdon D, Lee M, Legedza A, Paskavitz J, Prasad S, Richert J, Robbins A, Roberts S, Weiner H, Ramachandran R, Botfield M, De Jager PL (2015). Evaluating more naturalistic outcome measures. Neurol Neuroimmunol Neuroinflamm.

[18] Newland P, Oliver B, Newland J, Thomas F (2019). Testing feasibility of a mobile application to monitor fatigue in people with multiple sclerosis. J Neurosci Nurs.

[19] Giunti G, Fernández EG, Zubiete ED, Rivera Romero O (2018). Supply and demand in mhealth apps for persons with multiple sclerosis: systematic search in app stores and scoping literature review. JMIR Mhealth Uhealth.

[20] Pratap A, Grant D, Vegesna A, Tummalacherla M, Cohan S, Deshpande C, Mangravite L, Omberg L (2020). Evaluating the utility of smartphone-based sensor assessments in persons with multiple sclerosis in the real-world using an app (elevateMS): observational, prospective pilot digital health study. JMIR Mhealth Uhealth.

[21] Van Geel Fanny, Geurts E, Abasıyanık Z, Coninx K, Feys P (2020). Feasibility study of a 10-week community-based program using the WalkWithMe application on physical activity, walking, fatigue and cognition in persons with Multiple Sclerosis. Mult Scler Relat Disord.

[22] D'hooghe M, Van Gassen Geert, Kos D, Bouquiaux O, Cambron M, Decoo D, Lysandropoulos A, Van Wijmeersch Bart, Willekens B, Penner I, Nagels G (2018). Improving fatigue in multiple sclerosis by smartphone-supported energy management: the MS TeleCoach feasibility study. Mult Scler Relat Disord.

[23] Giunti G, Mylonopoulou V, Romero OR (2018). More stamina, a gamified mhealth solution for persons with multiple sclerosis: research through design. JMIR Mhealth Uhealth.

[24] Gauthier SA, Glanz BI, Mandel M, Weiner HL (2006). A model for the comprehensive investigation of a chronic autoimmune disease: the multiple sclerosis CLIMB study. Autoimmun Rev.

[25] Amtmann D, Bamer AM, Noonan V, Lang N, Kim J, Cook KF (2012). Comparison of the psychometric properties of two fatigue scales in multiple sclerosis. Rehabil Psychol.

[26] Cella D, Lai JS, Nowinski CJ, Victorson D, Peterman A, Miller D, Bethoux F, Heinemann A, Rubin S, Cavazos JE, Reder AT, Sufit R, Simuni T, Holmes GL, Siderowf A, Wojna V, Bode R, McKinney N, Podrabsky T, Wortman K, Choi S, Gershon R, Rothrock N, Moy C (2012). Neuro-QOL: brief measures of health-related quality of life for clinical research in neurology. Neurology.

[27] Actigraphy for sleep, chronobiology and physical activity. MotionWatch 8.

[28] Nox T3 HST. Nox Medical.

[29] Mobilengine. http://www.mobilengine.com/.

[30] Krupp LB, LaRocca NG, Muir-Nash J, Steinberg AD (1989). The fatigue severity scale. Application to patients with multiple sclerosis and systemic lupus erythematosus. Arch Neurol.

[31] Pedrelli P, Blais MA, Alpert JE, Shelton RC, Walker RSW, Fava M (2014). Reliability and validity of the Symptoms of Depression Questionnaire (SDQ). CNS Spectr.

[32] Johns MW (1991). A new method for measuring daytime sleepiness: the Epworth sleepiness scale. Sleep.

[33] Godin G, Shephard RJ (1985). A simple method to assess exercise behavior in the community. Can J Appl Sport Sci.

[34] Carver CS, White TL (1994). Behavioral inhibition, behavioral activation, and affective responses to impending reward and punishment: the BIS/BAS Scales. J Pers Soc Psychol.

[35] Hicks CL, von Baeyer Carl L, Spafford PA, van Korlaar Inez, Goodenough B (2001). The Faces Pain Scale-Revised: toward a common metric in pediatric pain measurement. Pain.

[36] Téllez N, Río J, Tintoré M, Nos C, Galán I, Montalban X (2005). Does the Modified Fatigue Impact Scale offer a more comprehensive assessment of fatigue in MS?. Mult Scler.

[37] Ahearn EP (1997). The use of visual analog scales in mood disorders: a critical review. J Psychiatr Res.

[38] Gur-Ozmen S, Leibetseder A, Cock HR, Agrawal N, von Oertzen TJ (2017). Screening of anxiety and quality of life in people with epilepsy. Seizure.

[39] Kim SH, Kim YH, Kim HJ (2015). Laughter and stress relief in cancer patients: a pilot study. Evid Based Complement Alternat Med.

[40] Fadaizadeh L, Emami H, Samii K (2009). Comparison of visual analogue scale and faces rating scale in measuring acute postoperative pain. Arch Iran Med.

[41] Malviya S, Polaner D, Berde C (2009). Acute pain. A Practice of Anesthesia for Infants and Children (Fourth Edition).

[42] Perski O, Blandford A, West R, Michie S (2017). Conceptualising engagement with digital behaviour change interventions: a systematic review using principles from critical interpretive synthesis. Transl Behav Med.

[43] O'Donnell D, Silva EJ, Münch M, Ronda JM, Wang W, Duffy JF (2009). Comparison of subjective and objective assessments of sleep in healthy older subjects without sleep complaints. J Sleep Res.

[44] Kurtzke JF (1983). Rating neurologic impairment in multiple sclerosis: an expanded disability status scale (EDSS). Neurology.

[45] Shin G, Feng Y, Jarrahi MH, Gafinowitz N (2019). Beyond novelty effect: a mixed-methods exploration into the motivation for long-term activity tracker use. JAMIA Open.

[46] Giunti G, Kool J, Romero OR, Zubiete ED (2018). Exploring the specific needs of persons with multiple sclerosis for mhealth solutions for physical activity: mixed-methods study. JMIR Mhealth Uhealth.

[47] Van Kessel Kirsten, Babbage DR, Reay N, Miner-Williams WM, Kersten P (2017). Mobile technology use by people experiencing multiple sclerosis fatigue: survey methodology. JMIR Mhealth Uhealth.

[48] Polhemus AM, Novák J, Ferrao J, Simblett S, Radaelli M, Locatelli P, Matcham F, Kerz M, Weyer J, Burke P, Huang V, Dockendorf MF, Temesi G, Wykes T, Comi G, Myin-Germeys I, Folarin A, Dobson R, Manyakov NV, Narayan VA, Hotopf M (2020). Human-centered design strategies for device selection in mhealth programs: development of a novel framework and case study. JMIR Mhealth Uhealth.

[49] Flachenecker P, Kümpfel T, Kallmann B, Gottschalk M, Grauer O, Rieckmann P, Trenkwalder C, Toyka KV (2002). Fatigue in multiple sclerosis: a comparison of different rating scales and correlation to clinical parameters. Mult Scler.

[50] Palotai M, Mike A, Cavallari M, Strammer E, Orsi G, Healy BC, Schregel K, Illes Z, Guttmann CR (2018). Changes to the septo-fornical area might play a role in the pathogenesis of anxiety in multiple sclerosis. Mult Scler.

[51] Ive P, MacLeod W, Mkumla N, Orrell C, Jentsch U, Wallis CL, Stevens W, Wood R, Sanne I, Bhattacharya D (2013). Low prevalence of liver disease but regional differences in HBV treatment characteristics mark HIV/HBV co-infection in a South African HIV clinical trial. PLoS One.

[52] Marrie RA, Reingold S, Cohen J, Stuve O, Trojano M, Sorensen PS, Cutter G, Reider N (2015). The incidence and prevalence of psychiatric disorders in multiple sclerosis: a systematic review. Mult Scler.

[53] Feinstein A, Magalhaes S, Richard JF, Audet B, Moore C (2014). The link between multiple sclerosis and depression. Nat Rev Neurol.

[54] Beck AT, Ward CH, Mendelson M, Mock J, Erbaugh J (1961). An inventory for measuring depression. Arch Gen Psychiatry.

[55] Chaudhuri A, Behan PO (2004). Fatigue in neurological disorders. The Lancet.

[56] Dobryakova E, DeLuca J, Genova HM, Wylie GR (2013). Neural correlates of cognitive fatigue: cortico-striatal circuitry and effort–reward imbalance. J Int Neuropsychol Soc.

[57] Asano M, Finlayson ML (2014). Meta-analysis of three different types of fatigue management interventions for people with multiple sclerosis: exercise, education, and medication. Mult Scler Int.

[58] Chaudhuri A, Behan PO (2000). Fatigue and basal ganglia. J Neurol Sci.

[59] Pardini M, Capello E, Krueger F, Mancardi G, Uccelli A (2013). Reward responsiveness and fatigue in multiple sclerosis. Mult Scler.

[60] Palotai M, Small C, Makris N, Somes NG, Pinzon AM, Rathi Y, Marzullo A, Levitt JJ, Bakshi R, Chitnis T, Guttmann CRG (2021). Microstructural changes in the left mesocorticolimbic pathway are associated with the comorbid development of fatigue and depression in multiple sclerosis. J Neuroimaging.

