# Supplementary material for: Usability of a Mobile App for Real-Time Assessment of Fatigue and Related Symptoms in Patients With Multiple Sclerosis: Observational Study
Source: JMIR Mhealth Uhealth. 2021 Apr 16;9(4):e19564. doi: 10.2196/19564 (PMC8087974; doi:10.2196/19564)
Supplement: Multimedia Appendix 1 [file mhealth_v9i4e19564_app1.pdf]

FATIGUE SEVERITY SCALE (FSS)

Date ______________________________ Name __________________________________

Please circle the number between 1 and 7 which you feel best fits the following statements. This refers to your usual way of life within the last week. 1 indicates "strongly disagree" and 7 indicates "strongly agree."

Read and circle a number.	Strongly Disagree → Strongly Agree						
1. My motivation is lower when I am fatigued.	1	2	3	4	5	6	7
2. Exercise brings on my fatigue.	1	2	3	4	5	6	7
3. I am easily fatigued.	1	2	3	4	5	6	7
4. Fatigue interferes with my physical functioning.	1	2	3	4	5	6	7
5. Fatigue causes frequent problems for me.	1	2	3	4	5	6	7
6. My fatigue prevents sustained physical functioning.	1	2	3	4	5	6	7
7. Fatigue interferes with carrying out certain duties and responsibilities.	1	2	3	4	5	6	7
8. Fatigue is among my most disabling symptoms.	1	2	3	4	5	6	7
9. Fatigue interferes with my work, family, or social life.	1	2	3	4	5	6	7

Fatigue

Please respond to each question or statement by marking one box per row.

	**In the past 7 days…**	**Never**	**Rarely**	**Sometimes**	**Often**	**Always**
NQFTG13	I felt exhausted…………………………...	☐ 1	☐ 2	☐ 3	☐ 4	☐ 5
NQFTG11	I felt that I had no energy………………...	☐ 1	☐ 2	☐ 3	☐ 4	☐ 5
NQFTG15	I felt fatigued……………………………..	☐ 1	☐ 2	☐ 3	☐ 4	☐ 5
NQFTG06	I was too tired to do my household chores.	☐ 1	☐ 2	☐ 3	☐ 4	☐ 5
NQFTG07	I was too tired to leave the house………...	☐ 1	☐ 2	☐ 3	☐ 4	☐ 5
NQFTG10	I was frustrated by being too tired to do the things I wanted to do…………………	☐ 1	☐ 2	☐ 3	☐ 4	☐ 5
NQFTG14	I felt tired…………………………………	☐ 1	☐ 2	☐ 3	☐ 4	☐ 5
NQFTG02	I had to limit my social activity because I was tired…………………………………	☐ 1	☐ 2	☐ 3	☐ 4	☐ 5
NQFTG01	I needed help doing my usual activities because of my fatigue……………………	☐ 1	☐ 2	☐ 3	☐ 4	☐ 5
NQFTG03	I needed to sleep during the day…………	☐ 1	☐ 2	☐ 3	☐ 4	☐ 5
NQFTG04	I had trouble <u>starting</u> things because I was too tired…………………………………..	☐ 1	☐ 2	☐ 3	☐ 4	☐ 5
NQFTG05	I had trouble <u>finishing</u> things because I was too tired……………………………...	☐ 1	☐ 2	☐ 3	☐ 4	☐ 5
NQFTG08	I was too tired to take a short walk………	☐ 1	☐ 2	☐ 3	☐ 4	☐ 5
NQFTG09	I was too tired to eat…………………......	☐ 1	☐ 2	☐ 3	☐ 4	☐ 5
NQFTG12	I was so tired that I needed to rest during the day……………………………………	☐ 1	☐ 2	☐ 3	☐ 4	☐ 5
NQFTG16	I felt weak all over………………………..	☐ 1	☐ 2	☐ 3	☐ 4	☐ 5

©2008-2013 David Cella and the PROMIS Health Organization on behalf of the National Institute for Neurological Disorders and Stroke (NINDS). Used with permission.

	In the past 7 days…	Never	Rarely	Sometimes	Often	Always
NQFTG17	I needed help doing my usual activities because of weakness……………………....	☐ 1	☐ 2	☐ 3	☐ 4	☐ 5
NQFTG18	I had to limit my social activity because I was physically weak……………………....	☐ 1	☐ 2	☐ 3	☐ 4	☐ 5
NQFTG20	I had to force myself to get up and do things because I was physically too weak..	☐ 1	☐ 2	☐ 3	☐ 4	☐ 5

©2008-2013 David Cella and the PROMIS Health Organization on behalf of the National Institute for Neurological Disorders and Stroke (NINDS). Used with permission.

Depression

Please respond to each question or statement by marking one box per row.

	**In the past 7 days…**	**Never**	**Rarely**	**Sometimes**	**Often**	**Always**
NQDEP13	I felt depressed…………………………..	☐ 1	☐ 2	☐ 3	☐ 4	☐ 5
NQDEP23	I felt hopeless……………………………	☐ 1	☐ 2	☐ 3	☐ 4	☐ 5
NQDEP07	I felt that nothing could cheer me up…….	☐ 1	☐ 2	☐ 3	☐ 4	☐ 5
NQDEP27	I felt that my life was empty……………...	☐ 1	☐ 2	☐ 3	☐ 4	☐ 5
NQDEP02	I felt worthless……………………………	☐ 1	☐ 2	☐ 3	☐ 4	☐ 5
NQDEP19	I felt unhappy……………………………	☐ 1	☐ 2	☐ 3	☐ 4	☐ 5
NQDEP21	I felt I had no reason for living…………..	☐ 1	☐ 2	☐ 3	☐ 4	☐ 5
NQDEP24	I felt that nothing was interesting………..	☐ 1	☐ 2	☐ 3	☐ 4	☐ 5
NQDEP04	I felt helpless…………………………..	☐ 1	☐ 2	☐ 3	☐ 4	☐ 5
NQDEP11	I felt that I wanted to give up on everything……………………………...	☐ 1	☐ 2	☐ 3	☐ 4	☐ 5
NQDEP03	I felt that I had nothing to look forward to.	☐ 1	☐ 2	☐ 3	☐ 4	☐ 5
NQDEP05	I withdrew from other people…………….	☐ 1	☐ 2	☐ 3	☐ 4	☐ 5
NQDEP06	I felt that everything I did was an effort….	☐ 1	☐ 2	☐ 3	☐ 4	☐ 5
NQDEP08	I was critical of myself for my mistakes…	☐ 1	☐ 2	☐ 3	☐ 4	☐ 5
NQDEP10	I felt sad………………………………..	☐ 1	☐ 2	☐ 3	☐ 4	☐ 5
NQDEP12	I felt lonely………………………………	☐ 1	☐ 2	☐ 3	☐ 4	☐ 5

©2008-2013 David Cella and the PROMIS Health Organization on behalf of the National Institute for Neurological Disorders and Stroke (NINDS). Used with permission.

	In the past 7 days…	Never	Rarely	Sometimes	Often	Always
NQDEP14	I felt discouraged about the future………..	☐ 1	☐ 2	☐ 3	☐ 4	☐ 5
NQDEP18	I found that things in my life were overwhelming…………………………….	☐ 1	☐ 2	☐ 3	☐ 4	☐ 5
NQDEP20	I felt unloved……………………………..	☐ 1	☐ 2	☐ 3	☐ 4	☐ 5
NQDEP25	I felt pessimistic………………………….	☐ 1	☐ 2	☐ 3	☐ 4	☐ 5
NQDEP26	I had trouble keeping my mind on what I was doing…………………………………	☐ 1	☐ 2	☐ 3	☐ 4	☐ 5
NQDEP28	I felt emotionally exhausted……………...	☐ 1	☐ 2	☐ 3	☐ 4	☐ 5
NQDEP29	I felt like I needed help for my depression.	☐ 1	☐ 2	☐ 3	☐ 4	☐ 5
NQDEP30	I had trouble enjoying things that I used to enjoy……………………………………..	☐ 1	☐ 2	☐ 3	☐ 4	☐ 5

©2008-2013 David Cella and the PROMIS Health Organization on behalf of the National Institute for Neurological Disorders and Stroke (NINDS). Used with permission.

Anxiety

Please respond to each question or statement by marking one box per row.

	**In the past 7 days…**	**Never**	**Rarely**	**Sometimes**	**Often**	**Always**
NQANX26	I felt uneasy………………………………	☐ 1	☐ 2	☐ 3	☐ 4	☐ 5
NQANX22	I felt nervous……………………………..	☐ 1	☐ 2	☐ 3	☐ 4	☐ 5
NQANX23	Many situations made me worry…………	☐ 1	☐ 2	☐ 3	☐ 4	☐ 5
NQANX20	My worries overwhelmed me…………....	☐ 1	☐ 2	☐ 3	☐ 4	☐ 5
NQANX27	I felt tense………………………………..	☐ 1	☐ 2	☐ 3	☐ 4	☐ 5
NQANX28	I had difficulty calming down.………….	☐ 1	☐ 2	☐ 3	☐ 4	☐ 5
NQANX09	I had sudden feelings of panic……………	☐ 1	☐ 2	☐ 3	☐ 4	☐ 5
NQANX07	I felt nervous when my normal routine was disturbed ……………………………	☐ 1	☐ 2	☐ 3	☐ 4	☐ 5
NQANX02	I felt fearful about my future……………..	☐ 1	☐ 2	☐ 3	☐ 4	☐ 5
NQANX03	I felt anxious……………………………..	☐ 1	☐ 2	☐ 3	☐ 4	☐ 5
NQANX04	I worried about my physical health………	☐ 1	☐ 2	☐ 3	☐ 4	☐ 5
NQANX05	I felt like I needed help for my anxiety.….	☐ 1	☐ 2	☐ 3	☐ 4	☐ 5
NQANX11	I was easily startled………………………	☐ 1	☐ 2	☐ 3	☐ 4	☐ 5
NQANX12	I felt fidgety……………………………..	☐ 1	☐ 2	☐ 3	☐ 4	☐ 5
NQANX13	I felt something awful would happen…….	☐ 1	☐ 2	☐ 3	☐ 4	☐ 5
NQANX14	I felt worried……………………………..	☐ 1	☐ 2	☐ 3	☐ 4	☐ 5

©2008-2013 David Cella and the PROMIS Health Organization on behalf of the National Institute for Neurological Disorders and Stroke (NINDS). Used with permission.

	In the past 7 days…	Never	Rarely	Sometimes	Often	Always
NQANX17	I suddenly felt scared for no reason………	☐ 1	☐ 2	☐ 3	☐ 4	☐ 5
NQANX18	I worried about dying……...……………	☐ 1	☐ 2	☐ 3	☐ 4	☐ 5
NQANX21	I felt shy…………………...……………	☐ 1	☐ 2	☐ 3	☐ 4	☐ 5
NQANX24	I had difficulty sleeping…….………….…	☐ 1	☐ 2	☐ 3	☐ 4	☐ 5
NQANX25	I had trouble relaxing…………………….	☐ 1	☐ 2	☐ 3	☐ 4	☐ 5

©2008-2013 David Cella and the PROMIS Health Organization on behalf of the National Institute for Neurological Disorders and Stroke (NINDS). Used with permission.

Sleep Disturbance – Short Form

Please respond to each question or statement by marking one box per row.

	In the past 7 days…	Never	Rarely	Sometimes	Often	Always
NQSLP02	I had to force myself to get up in the morning………………………………..	☐ 1	☐ 2	☐ 3	☐ 4	☐ 5
NQSLP03	I had trouble stopping my thoughts at bedtime………………………………...	☐ 1	☐ 2	☐ 3	☐ 4	☐ 5
NQSLP04	I was sleepy during the daytime…………	☐ 1	☐ 2	☐ 3	☐ 4	☐ 5
NQSLP05	I had trouble sleeping because of bad dreams…………………………………	☐ 1	☐ 2	☐ 3	☐ 4	☐ 5
NQSLP07	I had trouble falling asleep………………	☐ 1	☐ 2	☐ 3	☐ 4	☐ 5
NQSLP12	Pain woke me up…………………………	☐ 1	☐ 2	☐ 3	☐ 4	☐ 5
NQSLP13	I avoided or cancelled activities with my friends because I was tired from having a bad night's sleep………………………….	☐ 1	☐ 2	☐ 3	☐ 4	☐ 5
NQSLP18	I felt physically tense during the middle of the night or early morning hours………..	☐ 1	☐ 2	☐ 3	☐ 4	☐ 5

©2008-2013 David Cella and the PROMIS Health Organization on behalf of the National Institute for Neurological Disorders and Stroke (NINDS). Used with permission.

Modified Fatigue Impact Scale (MFIS)

Following is a list of statements that describe how fatigue may affect a person. Fatigue is a feeling of physical tiredness and lack of energy that many people experience from time to time. In medical conditions like MS, feelings of fatigue can occur more often and have a greater impact than usual. Please read each statement carefully, and then circle the one number that best indicates how often fatigue has affected you in this way during the past 4 weeks. (If you need help in marking your responses, tell the interviewer the number of the best response.) Please answer every question. If you are not sure which answer to select, please choose the one answer that comes closest to describing you. The interviewer can explain any words or phrases that you do not understand.

**Because of my fatigue during the past 4 weeks…**	**Never**	**Rarely**	**Sometimes**	**Often**	**Almost Always**
**1. I have been less alert.**	0	1	2	3	4
**2. I have had difficulty paying attention for long periods of time.**	0	1	2	3	4
**3. I have been unable to think clearly.**	0	1	2	3	4
**4. I have been clumsy and uncoordinated.**	0	1	2	3	4
**5. I have been forgetful.**	0	1	2	3	4
**6. I have had to pace myself in my physical activities.**	0	1	2	3	4
**7. I have been less motivated to do anything that requires physical effort.**	0	1	2	3	4

© 1994 National Multiple Sclerosis Society. Used with permission

**8. I have been less motivated to participate in social activities.**	0	1	2	3	4
**9. I have been limited in my ability to do things away from home.**	0	1	2	3	4
**10. I have had trouble maintaining physical effort for long periods.**	0	1	2	3	4
**11. I have had difficulty making decisions.**	0	1	2	3	4
**12. I have been less motivated to do anything that requires thinking.**	0	1	2	3	4
**13. My muscles have felt weak.**	0	1	2	3	4
**14. I have been physically uncomfortable.**	0	1	2	3	4
**15. I have had trouble finishing tasks that require thinking.**	0	1	2	3	4
**16. I have had difficulty organizing my thoughts when doing things at home or at work.**	0	1	2	3	4
**17. I have been less able to complete tasks that require physical effort.**	0	1	2	3	4
**18. My thinking has been slowed down.**	0	1	2	3	4
**19. I have had trouble concentrating.**	0	1	2	3	4
**20. I have limited my physical activities.**	0	1	2	3	4
**21. I have needed to rest more often or for longer periods.**	0	1	2	3	4

22. Which one of these items has the highest impact on your daily life activities? Please choose only one item:......

Reviewed by: ________________________________ *Title:* __________________ *Date:* ___________ *Time:* ________

© 1994 National Multiple Sclerosis Society. Used with permission

SELECTED QUESTIONS FROM

SYMPTOMS OF DEPRESSION QUESTIONNAIRE (SDQ)

Please answer all questions by circling the correct answer or the answer which seems the most appropriate to you.

Instructions: Please read each item and circle the number above the statement that you think applies to you. Some questions use the words "minimally," "moderately," "markedly," and "extremely." Minimally means that this item happens to you only rarely or that it is mild when it happens. Moderately means that this item bothers you some of the time but that it does not interfere with your life in any way. Markedly means that this item bothers you quite a bit and that it causes you some problems in your life. That is, it interferes with your ability to do certain things that are important to you such as working, taking care of your family, or enjoying time with friends. Extremely means that this problem troubles you a lot and that it interferes with your ability to do a lot of things.

28) How has your appetite been over the past month?

**1**	**2**	**3**	**4**	**5**	**6**
greater than normal	normal	minimally diminished	moderately diminished	markedly diminished	totally absent

29) Have you lost weight over the past month?

**1**	**2**	**3**	**4**	**5**	**6**
gained some weight	not at all	minimally	mildly	moderately	markedly

30) Has your appetite been excessive over the past month?

**1**	**2**	**3**	**4**	**5**	**6**
less	not at all	rarely	sometimes	frequently	all the time

31) Have you gained weight over the past month?

**1**	**2**	**3**	**4**	**5**	**6**
lost some weight	not at all	minimally	mildly	moderately	markedly

32) Have you had tachycardia/palpitations over the past month?

**1**	**2**	**3**	**4**	**5**	**6**
my heart rate felt slower than usual	not at all	rarely	sometimes	frequently	all the time

34) Have you had gastrointestinal (stomach or bowel) symptoms over the past month?

**1**	**2**	**3**	**4**	**5**	**6**
fewer symptoms than usual	not at all	rarely	sometimes	frequently	all the time

40) How has your sexual functioning been over the past month?

**1**	**2**	**3**	**4**	**5**	**6**
better than normal	normal	minimally diminished	moderately diminished	markedly diminished	totally absent

The Epworth Sleepiness Scale

The Epworth Sleepiness Scale is widely used in the field of sleep medicine as a subjective measure of a patient's sleepiness. The test is a list of eight situations in which you rate your tendency to become sleepy on a scale of 0, no chance of dozing, to 3, high chance of dozing. When you finish the test, add up the values of your responses. Your total score is based on a scale of 0 to 24. The scale estimates whether you are experiencing excessive sleepiness that possibly requires medical attention.

How Sleepy Are You?

How likely are you to doze off or fall asleep in the following situations? You should rate your chances of dozing off, not just feeling tired. Even if you have not done some of these things recently try to determine how they would have affected you. For each situation, decide whether or not you would have:

- No chance of dozing =0
- Slight chance of dozing =1
- Moderate chance of dozing =2
- High chance of dozing =3

Write down the number corresponding to your choice in the right hand column. Total your score below.

Situation	Chance of Dozing
Sitting and reading	•
Watching TV	•
Sitting inactive in a public place (e.g., a theater or a meeting)	•
As a passenger in a car for an hour without a break	•
Lying down to rest in the afternoon when circumstances permit	•
Sitting and talking to someone	•
Sitting quietly after a lunch without alcohol	•
In a car, while stopped for a few minutes in traffic	•

Total Score = ________________________

Analyze Your Score

Interpretation:

0-7: It is unlikely that you are abnormally sleepy.
8-9: You have an average amount of daytime sleepiness.
10-15: You may be excessively sleepy depending on the situation. You may want to consider seeking medical attention.
16-24: You are excessively sleepy and should consider seeking medical attention.

Reference: Johns MW. A new method for measuring daytime sleepiness: The Epworth Sleepiness Scale. *Sleep* 1991; 14(6):540-5.

Godin Leisure-Time Exercise Questionnaire

INSTRUCTIONS

In this excerpt from the Godin Leisure-Time Exercise Questionnaire, the individual is asked to complete a self-explanatory, brief four-item query of usual leisure-time exercise habits.

CALCULATIONS

For the first question, weekly frequencies of strenuous, moderate, and light activities are multiplied by nine, five, and three, respectively. Total weekly leisure activity is calculated in arbitrary units by summing the products of the separate components, as shown in the following formula:

Weekly leisure activity score = (9 × Strenuous) + (5 × Moderate) + (3 × Light)

The second question is used to calculate the frequency of weekly leisure-time activities pursued "long enough to work up a sweat" (see questionnaire).

EXAMPLE

Strenuous = 3 times/wk

Moderate = 6 times/wk

Light = 14 times/wk

Total leisure activity score = (9 × 3) + (5 × 6) + (3 × 14) = 27 + 30 + 42 = 99

Godin Leisure-Time Exercise Questionnaire

1. During a typical **7-Day period** (a week), how many times on the average do you do the following kinds of exercise for **more than 15 minutes** during your free time (write on each line the appropriate number).

Times Per Week

a) STRENUOUS EXERCISE
(HEART BEATS RAPIDLY)
(e.g., running, jogging, hockey, football, soccer, squash, basketball, cross country skiing, judo, roller skating, vigorous swimming, vigorous long distance bicycling) __________

MODERATE EXERCISE

(NOT EXHAUSTING) __________

(e.g., fast walking, baseball, tennis, easy bicycling, volleyball, badminton, easy swimming, alpine skiing, popular and folk dancing)

b) MILD EXERCISE
(MINIMAL EFFORT) __________

(e.g., yoga, archery, fishing from river bank, bowling, horseshoes, golf, snow-mobiling, easy walking)

2. During a typical **7-Day period** (a week), in your leisure time, how often do you engage in any regular activity **long enough to work up a sweat** (heart beats rapidly)?

OFTEN	SOMETIMES	NEVER/RARELY
1. |_|	2. |_|	3. |_|

Behavioral approach system/Behavioral avoidance system scales

Each item of this questionnaire is a statement that a person may either agree with or disagree with. For each item, indicate how much you agree or disagree with what the item says. Please respond to all the items; do not leave any blank. Choose only one response to each statement. Please be as accurate and honest as you can be. Respond to each item as if it were the only item. That is, don't worry about being "consistent" in your responses. Choose from the following four response options:

1 = very true for me
2 = somewhat true for me
3 = somewhat false for me
4 = very false for me

1. A person's family is the most important thing in life.
2. Even if something bad is about to happen to me, I rarely experience fear or nervousness.
3. I go out of my way to get things I want.
4. When I'm doing well at something I love to keep at it.
5. I'm always willing to try something new if I think it will be fun.
6. How I dress is important to me.
7. When I get something I want, I feel excited and energized.
8. Criticism or scolding hurts me quite a bit.
9. When I want something I usually go all-out to get it.
10. I will often do things for no other reason than that they might be fun.

11. It's hard for me to find the time to do things such as get a haircut.
12. If I see a chance to get something I want I move on it right away.
13. I feel pretty worried or upset when I think or know somebody is angry at me.
14. When I see an opportunity for something I like I get excited right away.
15. I often act on the spur of the moment.
16. If I think something unpleasant is going to happen I usually get pretty "worked up."
17. I often wonder why people act the way they do.
18. When good things happen to me, it affects me strongly.
19. I feel worried when I think I have done poorly at something important.
20. I crave excitement and new sensations.

21. When I go after something I use a "no holds barred" approach.
22. I have very few fears compared to my friends.
23. It would excite me to win a contest.
24. I worry about making mistakes.

Additional questions related to fatigue

1. Do you feel fatigued now? (Please select the appropriate.) yes/no

2. How often do you feel fatigued? (Please choose one)

Every day

Multiple times a week, but not every day

Multiple times a month, but not every week

I do not experience fatigue.

3. Usually at what time of day do you feel more fatigued? (You can choose more than one from the first 4 options regarding the mornings, afternoons, evenings and nights. If you always feel fatigued please select all 4 of these options.)

Mornings

Afternoons

Evenings

Nights

It varies

Never

4. Has your fatigue become more frequent or more intense in the past few years?

Yes

No

I do not experience fatigue.

5. Please estimate when you started experiencing fatigue: (If you have never experienced fatigue, please enter 0.) ……….. (Year):

6. In your opinion, is there anything that causes your fatigue? Please describe. (If you have never experienced fatigue, please enter no.)

…..………………………………………………………………………...

7. Is there anything that makes your fatigue worse? Please describe. (If you have never experienced fatigue, please enter no.)

……

8. Is there anything that alleviates your fatigue? Please describe. (If you have never experienced fatigue, please enter no.)

……

9/1. Did you experience fatigue before you were diagnosed with MS? Yes/No

9/2 If you experienced fatigue before you were diagnosed with MS, please estimate when this started? (If you have never experienced fatigue, please enter 0.)………………………………..…. (year)

9/3. Did you have any of the following complaints before you were diagnosed with MS? (Please select all appropriate answers)

Problems with memory

Problems with attention/concentration

Problems with decision-making

Problems with motivation

Sleepiness

Problems with sleep quality

Muscle weakness

Problems with physical activity

Social isolation

None of the above

Other: ……………………...……………………………………………………………………

…………………………………………………………………………………………………..

Physical Activity, Occupational Therapy, Psychological Therapy

1. How often do you do physical therapy? Please select the appropriate. If you are currently not under physical therapy, please choose the last answer.

every day
several times a week, but not every day
several times a month, but not every week
less than once a months

2. Please choose one of the following:

Physical activity alleviates my fatigue.
Physical activity makes my fatigue worse.
Physical activity alleviated my fatigue in the past, but now it has no effect on my fatigue.
Physical activity has no effect on my fatigue.
Currently I am not physically active, beyond limited activities of daily life (dressing, washing, eating, limited walking).

3. In the last year, have you been under occupational therapy (for example work simplification/energy conservation, nap schedule, use of assistive devices at home/work, cooling strategies/devices)? If yes, please write down the name of the occupational therapy. If no, please write "no" to this question.

…………………………………………………………….

4. How often do you do occupational therapy? Please select the appropriate. If you are currently not under occupational therapy, please choose the last answer.

every day
several times a week, but not every day
several times a month, but not every week
less than once a months

5. How does the occupational therapy influence your fatigue? Please choose the appropriate. If you are currently not under occupational therapy, please choose the last answer.

Occupational therapy improves my fatigue.
Occupational therapy makes my fatigue worse.
Occupational therapy alleviated my fatigue in the past, but now it has no effect on my fatigue.
Occupational therapy has no effect on my fatigue.
Currently, I am not under occupational therapy.

6. In the last year, have you been under any psychological treatment (such as psycho-dynamic therapy, cognitive-behavioral therapy, meditation, etc)? If yes, please write down the name of the psychological treatment. If you are not under any psychological treatment, please write "no" for this question.

……………………………………………………………..

7. How often do you receive psychological treatment? Please select the appropriate. If you are currently not under psychological treatment, please choose the last answer.

every day
several times a week, but not every day
several times a month, but not every week
less than once a months

8. How does the psychological treatment influence your fatigue? Please choose the appropriate. If you are currently not under psychological treatment, please choose the last answer.

Psychological treatment improves my fatigue.
Psychological treatment makes my fatigue worse.
Psychological treatment alleviated my fatigue in the past, but now it has no effect on my fatigue.
Psychological treatment has no effect on my fatigue.
Currently, I am not under psychological treatment.

Questions related to smoking, nicotine and caffeine consumption

1. When you feel fatigued, can smoking influence your fatigue? Please choose the appropriate answer. If you do not have fatigue or you do not smoke, please choose the last answer.

Smoking alleviates my fatigue.
Smoking aggravates my fatigue.
Smoking has no influence on my fatigue.
I don`t know.
Not applicable.

2. When you feel fatigued, can nicotine-containing patch or chewing gum influence your fatigue? Please choose the appropriate answer. If you do not have fatigue or you do not use nicotine-containing patch or chewing gum, please choose the last answer.

Nicotine alleviates my fatigue.
Nicotine aggravates my fatigue.
Nicotine has no influence on my fatigue.
I don`t know.
Not applicable.

3. When you feel fatigued, can caffeine-containing drinks (such as coffee, tea or coke) or caffeine pills influence your fatigue? If you do not have fatigue or you do not use caffeine-containing drinks or pills, please choose the last answer.

Caffeine alleviates my fatigue.
Caffeine aggravates my fatigue.
Caffeine has no influence on my fatigue.
I don`t know.
Not applicable.

Sleep diary

1. Final Wake time? ___________

2. Time out of bed? ________________
(if not at final wake time)

3. How long do you think it took you to fall asleep? ☐☐ : ☐☐ (Hours) (Minutes)

4. How many times did you wake up? ☐☐

5. How much sleep do you think you got? ☐☐ : ☐☐ (Hours) (Minutes)

---

6. How sound do you think your sleep was?

- ☐ Extremely light
- ☐ Very light
- ☐ Light
- ☐ Average
- ☐ Sound
- ☐ Very Sound
- ☐ Extremely Sound

7. How would you evaluate your sleep?

- ☐ Extremely bad
- ☐ Very bad
- ☐ Bad
- ☐ Average
- ☐ Good
- ☐ Very Good
- ☐ Extremely good

8. How sleepy do you feel right now?

- ☐ Not sleepy at all
- ☐ Slightly sleepy
- ☐ Somewhat sleepy
- ☐ Sleepy
- ☐ Moderately sleepy
- ☐ Very sleepy
- ☐ Extremely sleepy

9. How refreshed do you feel right now?

- ☐ Not refreshed at all
- ☐ Slightly refreshed
- ☐ Somewhat refreshed
- ☐ Refreshed
- ☐ Moderately refreshed
- ☐ Very refreshed
- ☐ Extremely refreshed

10. How tense do you feel right now?

- ☐ Not tense at all
- ☐ Slightly tense
- ☐ Somewhat tense
- ☐ Tense
- ☐ Moderately tense
- ☐ Very tense
- ☐ Extremely tense
